# The HERrespect intervention to address violence against female garment workers in Bangladesh: study protocol for a quasi-experimental trial

**DOI:** 10.1186/s12889-018-5442-5

**Published:** 2018-04-18

**Authors:** Mahfuz Al Mamun, Kausar Parvin, Marat Yu, Jessica Wan, Samantha Willan, Andrew Gibbs, Rachel Jewkes, Ruchira Tabassum Naved

**Affiliations:** 1 Health Systems and Population Studies Division, icddr,b, Dhaka, Bangladesh; 2 BSR, Hong Kong, China; 3 Gender and Health Research Unit, South African Medical Research Council, Pretoria, South Africa; 4 School of Public Health, University of the Witwatersrand, Johannesburg, South Africa

**Keywords:** Intimate partner violence, Workplace violence, Female garment worker, Bangladesh, Quasi-experimental trial

## Abstract

**Background:**

Women in Bangladesh experience high rates of Intimate Partner Violence (IPV). IPV is more prevalent against income earning women compared to their non-earning counterparts, and Workplace Violence (WPV) is also common. Such violence is a violation of women’s rights, and also constrains them from contributing to their personal growth, household, community and the economy at large. There is limited evidence on what works to prevent IPV and WPV amongst garment workers. This paper describes an evaluation of HERrespect, an intervention which aims to reduce IPV and WPV against female garment workers in and around Dhaka, Bangladesh.

**Methods:**

The trial employs a quasi-experimental design, with four intervention and four control factories. In the intervention factories a randomly selected cohort of married female line workers, a cohort of male line workers, and all middle management staff received the intervention. The intervention strategies involved (1) gender transformative group-based training for workers and management staff; (2) joint session between workers (15 female and male) and middle-management staff; (3) factory-wide activities; (4) awareness raising among top management; (5) factory policy review and development and 6) a community based campaign. For the evaluation, a cohort of randomly selected female workers and a cohort of selected management staff have been established. All workers (*n* = 800) and management staff (*n* = 395) from these cohorts were interviewed at baseline using two different questionnaires, and will be interviewed in the endline, 24 months post-baseline. Intention to treat analysis will be used for assessing the impact of HERrespect, comparing the intervention and control factories.

**Discussion:**

To our knowledge this is the first study that seeks to evaluate the impact on IPV and WPV, of group sessions with female workers, male workers, and management; factory-wide campaigns and a community intervention among female garment workers in Bangladesh. Apart from informing programmers and policy makers about intervention effectiveness in reducing IPV and WPV against female garment workers this study will also present evidence on an intervention tailored to the situation in the garment sector, which makes HERrespect scalable.

**Trial registration:**

ClinicalTrials.gov NCT03304015, retrospectively registered on October 06, 2017.

**Electronic supplementary material:**

The online version of this article (10.1186/s12889-018-5442-5) contains supplementary material, which is available to authorized users.

## Background and rationale

Women in Bangladesh experience high rates of Intimate Partner Violence (IPV). In recent research, 54% of the ever-married women reported lifetime physical and/or sexual IPV and 27% of them reported exposure to this violence during the last 12 months [1]. Common correlates of IPV in Bangladesh include young age, poverty, low education, early marriage, witnessing abuse of mother by father in the family, childhood experience of violence, dowry, and poor spousal communication [2–5].

Gender inequalities shaping women’s experiences of IPV are widespread in Bangladesh. Women in Bangladesh are expected to be subservient, while men expect to control women and their sexuality. The social structure enforces this control by imposing confinement to home, *purdah*, restrictions on interactions with males and limits on employment. These norms constrain women’s social and financial power and skills, while positioning men in their “superior” role. Violence against women and girls (VAWG) is widely accepted and used as a tool for punishment of any transgression of social norms, for controlling women, and upholding men’s honour and status [6–8].

Despite the restrictions on women’s engagement in public spaces, more women are now involved in income generating activities. Women’s labour force participation rate is about 36% in Bangladesh [9], where 12% percent of the working women are employed in the manufacturing sector including garment sector. The ready-made garment (RMG) sector is the main driver of economic growth and formal employment in Bangladesh. In 2015, it contributed to 82% of Bangladesh’s total exports, valued at over $34 billion [10]. Over 4.2 million, usually low skilled, workers are formally employed in more than 4500 factories [11]. Similar to other labour intensive and export-oriented industries in developing economies, the RMG sector offers employment opportunities for women, particularly notable in a country where women traditionally do not work outside the home [12]. Approximately 80% of workers in the RMG sector in Bangladesh are women [13].

There are different perspectives on the impact of employment on women in the garment sector. For some young women in Bangladesh, it has contributed to their empowerment and changed their lives. Due to access to garment sector jobs, some young women delay marriage and childbirth [14]. And with their own income they are more likely to make their own decisions on spending that takes account of their health, leading to improvements in their well-being and that of their family [15]. Overall, formal employment and bringing home earnings may raise female workers’ value within their own family and may improve their capacity to negotiate within their households. They may see improvements in their position in the household vis-à-vis other family members [12, 14–16].

However, women’s employment may also increase their risk of abuse and physical violence. The literature on factors associated with IPV shows that in some patriarchal contexts, working women may be more vulnerable to IPV [3, 6, 8, 17]. According to the 2015 Bangladesh National Violence against Women Survey a higher proportion (33%) of income earning women reported physical and/or sexual violence during the past 12 months than their non-earning counterparts (26%) [1]. Some authors have suggested that employment may result in more violence if women are perceived to be challenging social norms and men’s provider role through their income-generating activities [18]. In addition, waged work may also increase conflict if women are tired after work and perceived as deficient in the domestic tasks that they are expected to carry out without male assistance, if men are suspicious about their wives meeting other men at work, suspicious when they come home late or intermittently work overtime, and if working and earning makes them challenge aspects of the gender regime at home.

Very little is known about workplace violence (WPV) against working women in low income settings. According to the Report on 2015 National Violence against Women Survey in Bangladesh women perceived the workplace as the second most likely place to experience violence after the household [1]. WPV against female workers in garment factories is also understudied. Scanty literature shows that despite the positive impact of formal employment in the garment sector on women’s economic and social empowerment, female workers are likely to experience violence in the factories. Fair Wear Foundation found that 75% of workers had experienced verbal violence at work, 20% experienced physical violence, and 30% had experienced psychological violence [13]. Sixty percent of female garment workers had experienced sexual harassment in the factories [13]. Qualitative research suggests that severe emotional and economic violence is common in this sector, while physical and sexual violence is not uncommon [19]. Most common perpetrators of workplace violence are mid- and low-level factory management staff, the majority of whom are male. The most common forms of violence facing female garment workers include name-calling, shouting, yelling, teasing, rough behaviour, being referred to using slang, and propositioning [20].

In general, evidence on what works in addressing IPV and WPV against working women, particularly garment workers is limited worldwide. Given the massive increase in women’s employment in factories globally, and specifically in Bangladesh, with the potential to increase women’s experiences of violence in the home and workplace, there remains a lack of effective interventions to reduce this. Although there is evidence that a combination of economic empowerment and gender interventions reduces IPV effectively in other settings [21–23] there has been no attempt at measuring the effect of gender interventions among female garment workers, who are relatively economically empowered compared to their peers. This paper describes an evaluation of HERrespect, an intervention which aims to address IPV and WPV against female garment workers in and around Dhaka. This research is being conducted by icddr,b in collaboration with South African Medical Research Council (SAMRC) and the intervention is being implemented by BSR, Change Associates Limited and WE CAN. The study is being conducted under the DFID-funded global programme “What Works To Prevent Violence Against Women and Girls?”

## Methods

### Objectives of the study

The primary objectives of the study are to assess whether the HERrespect intervention reduces female garment workers’ experiences of: (i) physical, sexual, and physical and/or sexual intimate partner violence and (ii) workplace violence in Bangladesh.

Additionally, the study will assess whether HERrespect is effective in: (1) increasing gender equitable attitudes, self-esteem, and knowledge and uptake of services for addressing IPV among female garment workers; (2) reducing acceptance of VAWG and the rate of depression among female garment workers; (3) increasing gender equitable attitudes and knowledge regarding laws and policies addressing gender discrimination and workplace violence against women among management; and (4) improving management styles and attitudes towards workers among management.

### Trial design

The trial employs a quasi-experimental design. It is being conducted in four intervention and four control factories. In the intervention factories a randomly selected cohort of married female line workers, a cohort of male line workers, and all middle management staff received the main intervention. In addition, factory-wide campaigns were carried out, and a community activity in one intervention community. For the evaluation, two cohorts have been established and will be followed up over 24 months in each of the intervention and control factories. One is a cohort of randomly selected female line workers (100 per factory) and the second is a cohort of selected management staff with frequent interaction with workers (50 in each factory). Two different questionnaires are administered for workers and management staff (see Additional files 1 and 2). All the workers and management staff from these cohorts were interviewed at baseline, and will be interviewed in the endline. The baseline was conducted before the intervention started, while the endline will be conducted 24 months post-baseline.

### The intervention

HERrespect is a workplace programme developed by BSR and SAMRC, with inputs from Change Associates Limited. Design of HERrespect is guided by the programmes Theory of Change and the formative research conducted by icddr,b.

#### Theory of change

The base of the Theory of Change begins with a core problem that female workers in the RMG industry in Bangladesh are susceptible to high levels of violence, both in the workplace and their intimate relationships. There is a lack of critical awareness about gender and rights among workers, and violence against women (VAW) is normalized and generally accepted in both workplace and intimate relationships. The dominant social norms in Bangladesh and the disciplinary nature of operating a factory reinforce the submissive identity of female workers and the unequal relationship between managers and workers. Female workers also have poor skills in communicating with managers and intimate partners and lack effective coping mechanisms to deal with stress. Moreover, workers do not have sufficient information on their rights and available resources at work and in the community.

To overcome the barriers, a combination of intervention strategies have been adopted to: (1) develop assertive communication skills; (2) build an understanding of gender and power and how it interplays in relationships; (4) reaffirm that violence is never justified; (5) acknowledge the stress facing female workers; (6) inform female workers of service providers and support within the factory, and (7) inform female workers of factory policies and local laws.

The goal of HERrespect is to cultivate more gender equitable attitudes and relationships among women and men in the RMG industry in Bangladesh, which ultimately will contribute to preventing VAW at workplace and intimate relationships. After participating in the interventions, workers are expected to become more willing and equipped to engage in respectful dialogue with managers/colleagues at work and intimate partners. They will have greater gender awareness and have less acceptance of the use of violence. It is expected that workers will have better coping mechanisms for stress. Finally, they should have greater awareness of prevention strategies and support services for abused women inside and outside the workplace. The theory of change is presented in Table 1.

**Table 1:** HERrespect theory of change

Outcomes	Workers become more willing and equipped to engage in respectful dialogue with managers/colleagues at work and intimate partners	Greater gender awareness and sense of empowerment		Less acceptance and normalization of the use of violence	Workers have better coping mechanisms to work stress	Greater awareness on protection mechanism and support for abused women inside and outside the workplace	
Outputs	Workers are better able to communicate their needs at home and at work (i.e. asking for leave, explaining their needs, saying no in respectful ways)	Workers are more aware of how gender shapes their roles and responsibilities, and also how men can become more involved in care responsibilities.	Workers are more aware of how power affects their relationships and interactions with managers and their husbands	Workers can identify both joyful and harmful relationships, as well as ways to ameliorate them	Workers understand the sources of stress at work (collectively) and develop ways to deal with stress	Workers are aware of where to seek help inside and outside the factory	Workers aware of their rights and the responsibilities of the factory and the government
Exercises needed	Active listening; body language; attack, avoid, manipulate; I statements; saying no in respectful ways; assertive responses	Men and women: ideal and reality	Power over; statues of power	Joys and challenges; ways to get hurt; consequences of violence	Managing stress at work	Support systems for abused women; providing support as Change Makers	Factory policies and local laws
Inputs	Critical reflection on different ways of communicating (physical and verbal) through interactive activities	Understanding the impact of gender expectations (on both women and men) in their day-to-day lives	Reflecting on how power affects our relationships, how we communicate, how we make decisions	Sharing of experiences of violence at home, at work, in the communities, and its consequences	Sharing and mapping of the different points of stress at work	Explaining to workers the types of services (medical, legal, counseling, shelter) for abused women	Explaining to workers the factory policies and local laws
Responses	Develop assertive communication skills	Build an understanding of gender and how it impacts our roles and responsibilities	Build understanding of power and how it interplays in different relationships	Reaffirm that violence is never justified, no one deserves violence and everyone deserves respect in relationships	Acknowledge the stress facing female workers	Inform female workers of service providers and support within the factory	Inform female workers of factory policies and local laws
Barriers	Poor communication skills in speaking to managers/colleagues and intimate partners	Gender inequity plays out in the factory through male management dominance over female workers; at home, dominance of husbands over their wives		There is a general acceptance of the use of violence in relationships	Female workers are under constant stress and do not have proper coping mechanisms	Female workers have knowledge gaps - they are unaware of support services and their rights at work and outside of work	
Problem	Female workers are susceptible to violence both at work and at home. In the RMG setting, the use of violence is normalized, for instance, the use of name-calling and shouting. Female workers are at risk of sexual harassment. In Bangladesh, 73% of married women shared that they have experienced some form of violence by their partners in their lifetime.						

#### Intervention components

The HERrespect intervention consists of two components – a factory component and a community component. The factory component, which is the key focus of HERrespect was implemented by Change Associates Limited and the smaller community component was implemented by WE CAN. Better Work, an International Finance Corporation and International Labour Organization programme, provided technical advice on the development and review of factory’s violence and harassment policies. The intervention components in the workplace were:

1. Separate gender transformative training for female and male workers (single sex groups) and management staff of 18 h (six 3-h modules), were held over 10 months and delivered to groups of 25 people, one module per month. The session topics include communication skills (e.g. listening, body language, etc.); assertive responses; reflection and discussion of gender roles and norms, and relationships; power; violence in relationships (causes, consequences and support system); stress and conflict management; factory policy analysis; goal setting and being a Change Maker. The curriculum is participatory, taking reference from Stepping Stones and Freirean reflective pedagogy.
2. Joint sessions between selected workers (15 female and male) and middle-management staff (10), were held after the third, fifth and sixth group sessions.
3. Factory-wide activities and campaigns using factories public address systems, skits and plays and other behaviour change communication materials.
4. Awareness raising sessions among senior management (e.g., General Manager, Human Resources and Administration, Directors, etc), which were 2 h long.
5. Factory policy review and development. Activities included at least one meeting every two months with the Factory Well-being Committee. The topics discussed included: reviewing existing and developing new gender policies and mechanisms to prevent and address sexual harassment; designing and implementing factory-wide promotional activities/campaigns; reporting on progress and challenges to senior management on a regular basis; and designing and implementing sustainability plans.

The small community engagement component, implemented by WE CAN, complemented the workplace initiatives by targeting the members of one intervention community where female workers of a selected intervention factory live. Specifically, the community component aims to mobilize men in the communities, including husbands of female workers and community elders and leaders, to prevent and combat VAWG at home and communities. Its activities included a video screening in the community, a couples’ fair, door to door campaign, meetings of Change Makers (including HERrespect participants) and a national sharing meeting.

### Study setting

The study is being conducted in Dhaka, Gazipur and Narayanganj districts in and around Dhaka, Bangladesh. Five study factories were selected from Gazipur district and one was selected from the Dhaka export processing zone situated outside Dhaka city. The remaining two factories are in Narayanganj, one of these is from a Bangladesh small and cottage industries corporation zone.

### Trial outcomes

Considering the objectives of the HERrespect intervention, four primary outcomes, split into two groups, were identified:

1. Three primary outcomes are focused on: physical, sexual, and physical and/or sexual IPV experienced by female garment workers in the past 12 months:
  - The proportion of women experiencing any physical IPV from husband during the past 12 months.
  - The proportion of women experiencing any sexual IPV from husband during the past 12 months.
  - The proportion of women experiencing severe physical and/or sexual IPV from husband during the past 12 months.

These outcomes are being measured using a set of questions based on the World Health Organization (WHO) violence against women instruments, which are designed to minimize reporting biases that arise from subjective perceptions of abuse by asking only about specific behaviours perpetrated by a male partner. The questions have been adapted for the “What Works To Prevent Violence against Women and Girls?” Global Programme and are being used across the evaluations within the programme.

2. Violence witnessed or experienced at workplace in the past 4 weeks by female garment workers: This outcome is being assessed with an 8-item scale asked to the female garment workers, which was loosely developed as an adaptation of the peer victimisation scale [24]. The adaptation has been to measure ‘experiencing or witnessing’ victimisation as this was deemed to be less sensitive with factory management than actual reported victimisation. The adaptation of questions drew heavily on the findings of the formative research on the types of violence which occur in the work place.

The ten secondary outcomes are being assessed, reflecting hypothesised pathways of change leading to the primary outcomes. Four secondary outcomes focus on gender attitudes and responses to violence:

1. Acceptance of VAWG amongst female garment workers: Acceptance of VAWG among female garment workers is being measured using statements adapted from the Gender Equitable Men (GEM) scale [25] and WHO multi-country study on women’s health and domestic violence against women [26].
2. Gender equitable attitudes of management staff: Gender equitable attitude of the management staff is being measured using statements adapted from the GEM scale [25], WHO multi-country study on women’s health and domestic violence against women [26] and the South African study on men, masculinities, violence and HIV [27].
3. Response to IPV amongst female garment workers: A single item explores women’s help seeking in response to IPV. The question heavily drew upon the WHO multi-country study questionnaire [26] and the SAFE questionnaire [28]. This question will only be asked to those who experienced physical and/or sexual violence during last 12 months.
4. Knowledge of services in response to IPV amongst female garment workers: One question assesses the knowledge of the workers about where to seek help after experiencing IPV.

Two secondary measures assess women’s well-being:

5. Self-esteem of female garment workers: The Rosenberg Self-Esteem scale is being used to measure self-esteem [29]. It measures global self-worth by measuring both positive and negative feelings about self. The scale is uni-dimensional and has shown strong convergent validity for men and women from different ethnic groups [30].
6. Depression among female garment workers: Depression in the past week is being measured using the Center for Epidemiologic Studies Depression (CES-D) scale used widely in different settings (including Bangladesh) and showing high reliability and validity [31]. Each question is a statement and asked the respondent to answer how many days she has had particular feelings or ideas.

Four secondary outcomes focus specifically on management knowledge, practices and experiences:

7. Management style: An adapted version of the social power scale developed by French and Raven (1959) and revised by Swasy (1979) is being asked to the female garment workers for measuring management styles of the managers [32, 33].
8. Knowledge regarding laws and policies: The management staff are being asked five questions for assessing knowledge of the management staff on gender rights of women and protection of women against IPV and workplace violence guaranteed in laws and policies of Bangladesh.
9. Attitudes regarding laws and policies: The management staff are being asked five questions to measure their attitudes regarding the existing laws and policies about protection of women against spousal violence and workplace violence.
10. Burn out among management staff: The Maslach Burnout Inventory-Human Service Survey (MBI-HSS) is being adapted for measuring burnout of the management staff in the studied garment factories. The MBI-HSS has four subscales, which are Emotional Exhaustion (nine items); Personal Accomplishment (eight items); Depersonalization (five items); and three Optional items. Together, the subscales of the MBI-HSS provide a three dimensional perspective on burnout. Various studies regarding the MBI-HSS confirmed the soundness of the psychometric properties of the instrument (including variance of factor loadings), as well as its reliability and validity [34].

The summary of the trial outcomes is presented in Table 2.

**Table 2:** Primary and secondary outcomes of the trial

	Typical Item	Response categories	Number of items	Cronbach Alpha for Worker data	Cronbach Alpha for Management data	Method of scaling	Hypothesis regarding expected change
Primary outcomes							
Physical IPV during past 12 months	In the past 12 months, how many times your current husband has slapped you or something to you that could hurt you?	Never, once, few, many	5	–	–	Binary - never compared to once or more	Decrease
Sexual IPV during past 12 months	In the past 12 months, how many times your current husband has physically forced you to have sexual intercourse when you did not want to?	Never, once, few, many	5	–	–	Binary - never compared to once or more	Decrease
Severe physical and/or sexual IPV during past 12 months	Questions asked for measuring physical and sexual violence during past 12 months	Never, once, few, many	10	–	–	Binary - never compared to once or more	Decrease
Witnessed or experienced workplace violence	In the past four weeks, how often have you experienced or witnessed a manager call a worker names?	Never, once, 2-3 times, many times	8	0.83		Mean	Decrease
Secondary outcomes							
Gender attitudes and responses to violence							
Acceptance of VAWG among female workers	In your opinion, does a man have good reason to hit his wife if she disobeys him?	4-point Likert: Strongly disagree, disagree, agree, strongly agree	6	0.69	–	Mean	Decrease
Positive gender attitudes among management staff	I think that a woman needs her husband’s permission to do paid work.	4-point Likert: Strongly disagree, disagree, agree, strongly agree	13	–	0.68	Mean	Decrease
Response to IPV	Did you go to any person/place for help when you experienced any physical or sexual violence during last 12 months?	Yes, no	1	–	–	Binary – yes compared to no	Increase
Knowledge of services in response to IPV	Do you know from where one can get help for such experience? If yes, where?	Yes, no	1	–	–	Binary – yes compared to no	Increase
Women’s well-being							
Self-esteem of the female garment workers	At times, You think you are no good at all.	4-point Likert: Strongly disagree, disagree, agree, strongly agree	10	0.76	–	Mean	Increase
Depression among female garment workers during past week	During the past week you thought your life had been a failure.	Rarely or none of the time; some or a little of the time; moderate amount of time; most or all of the time	13	0.87	–	Mean	Decrease
Management knowledge, practices and experiences							
Better management style of management staff	If you do not comply with your supervisor, you will not be rewarded	Very unlikely; Somewhat likely; Very likely	14	0.90	–	Mean	Increase
Correct knowledge regarding laws and policies	Are there laws/policies in this country that protect women from spousal violence?	Yes; no; don’t know	5	–	–	% correct compared to % incorrect	Increase
Positive attitudes regarding laws and policies	To your opinion - “Sexual remark and gesture is an act of workplace violence”	Strongly agree; agree; no opinion; disagree; strongly disagree	5	–		Mean	Increase
Burn out among management staff	You feel used up at the end of the workday	Never; A few times year; Monthly; A few times a month; Every Week; A few times a week; Everyday	16	–	0.67	Mean	Decrease

### Sample size

For the worker survey, the sample size calculation was based on the primary outcome – physical IPV against female garment workers. Expecting 56% baseline prevalence of past year physical IPV experienced by the female garment workers (icddr,b unpublished data), and anticipating a 20% effect size, 5% level of significance and 80% power, the required sample size was 330 workers for both the intervention and control groups. To account for a 20% lost to follow up the sample size increased to 396 workers for both the groups, which was rounded up to 400. Thus, a sample of 100 workers would be required from each of the intervention and control factories, giving 800 in total.

For the management survey, the sample size calculation was based on gender inequitable attitudes among garment management staff. Anticipating 50% baseline prevalence of high or moderate gender equitable attitudes among the management staff, a 30% effect size, 5% level of significance and 80% power, the required sample size was 183 management staff for both intervention and control groups. Anticipating 10% loss to follow up, the sample size increased to 200 for both the groups. Thus a sample of 50 management staff would be required from each of the intervention and control factories, giving 400 in total.

### Eligibility criteria

Workers were eligible for the research if they were currently married and living with husband (necessary for measuring IPV) and had been working in the current factory for at least one year. All management staff in the factory management were eligible to participate in the study.

### Participant timeline

**Fig. 1:**
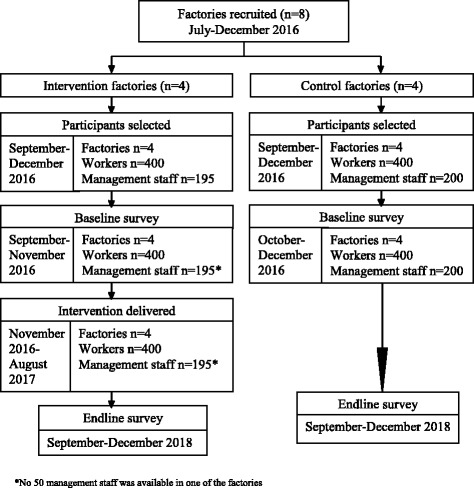
Participant timeline

Recruitment of the factories was completed over a 6-month period from July to December, 2016. The study participants (workers and management staff) were recruited and the baseline data were collected during September to December, 2016. As soon as a factory was recruited baseline started in that factory while recruitment was continuing for other factories. Implementation of the intervention in the first intervention factory was started in November, 2016 once the survey was completed for that factory, and subsequently in other factories. The intervention was completed in August, 2017. The endline will be conducted from September to November 2018 (See Fig. 1).

### Recruitment and allocation

BSR works with a network of international brands and buyers via HERproject, a women empowerment initiative in the supply chain, in Bangladesh, and both intervention and control factories in HERrespect were recruited through buyers as per the selection criteria, through one-on-one engagement and a suppliers’ workshop. Four buyers participated in nominating eight factories (four intervention and four control) to join HERrespect. Participant factories had strict preference for being included as an intervention factory or control factory and were recruited on that basis. Care was taken to select control factories at a distance from the intervention factories to prevent potential contamination. Also, preference was given to factories that had not recently completed similar training programs or interventions in gender-based violence.

Each factory provided a self-selected list of 315 workers from their full list of workers. A listing was then carried out of these selected 315 workers within each factory mainly for the purpose of screening eligibility. Once eligibility had been established and agreement to participate given, 100 workers were randomly selected from each factory. The workers from the intervention factories were then assigned to group sessions and interviewed at baseline. If more than one worker belonged to the same household, one was chosen randomly for participating in the study. Each factory provided a self-selected list of 50 management staff having direct interaction with workers. This cohort of management staff are interviewed at baseline and endline. However, all management staff received the group sessions.

### Blinding

No one was blinded to their study arm allocation.

### Listing of workers

The listing of workers was carried out by eight trained female interviewers in each factory. A researcher from icddr,b and a designated person from the factory worked closely with the listing team. The factory provided space for conducting the listing interviews and allowed the workers to give 5 min for an interview to determine eligibility for the study.

### Baseline survey

#### Survey team, training and selection

The worker survey team consisted of 12 female interviewers, three female supervisors, three male field assistants, and one survey co-ordinator. The management survey team consisted of 6 male interviewers and one male supervisor. One quality control officer (QCO) was deployed for the surveys, and interviewed female management staff. One research officer was responsible for overseeing both the surveys. Shortlisted interviewer candidates for the worker survey received a 12-day training and the shortlisted interviewer candidates for the management survey received an 8-day participatory training on gender, violence against women, ethics, survey methods, the questionnaire, and use of tablets. The training included role play, pair practice, mock interview and discussion on survey techniques. A field test was included as part of the training and evaluation. The survey team members were finally selected based on their performance during the training and piloting.

#### Data collection tools

The questionnaire for worker and management surveys were developed in English and then translated into Bengali. The Bengali version of the worker questionnaire was pre-tested with 8 female garment workers and the management questionnaire was pre-tested with 10 garment management staff. Cognitive pre-testing was carried out for sections that had not been previously used in Bangladesh. The cognitive pre-test helped us in identifying sections and questions participants found difficult to understand and to adapt as appropriate. A pilot with 40 workers and 15 management staff was also conducted. The feedback received was incorporated into the questionnaire.

#### Data collection methods

Baseline data were collected between September to December 2016 using face-to-face interviews, with Personalized Digital Assistants (PDAs) to reduce data entry error as logic checks were built in. Workers’ interviews were conducted in private in a location convenient for participants outside the factory. According to the study participants’ choice 98% of workers were interviewed at their own home and 2% of them were interviewed outside of their home (e.g., relatives or neighbours home, Non-Government Organisation (NGO) office, etc.). The interviews took place in the evening after work during work days and in the day time during weekends and holidays. The usual time of the interviews was between 7 and 10 pm on work days and 9 am to 4 pm on weekends and holidays. The interviewers phoned the participants a day before the target interview date to schedule the interview and obtain directions for the interview venue. The interviews with the management staff were conducted in private within the factory during office hours. The appointments were arranged in advance.

Workers and management staff who were not available to be interviewed during baseline data collection were replaced. The replacement of a worker was done by randomly selecting another eligible worker from the same factory and the replacement of a management staff was done by the factory itself. But no replacement was made after the data collection had been completed.

#### Data quality monitoring and management

During fieldwork for the worker survey each interviewer was accompanied to the interview venue either by a supervisor or a field assistant. The supervisor and the field assistant made sure that the interview could be started in private. Once all the interviews started, the supervisor and the field assistant made rounds to check whether the interviews were uninterrupted. They also helped, if needed, to handle gatekeepers. The survey coordinator randomly visited the survey teams for spot checks.

The first level monitoring was performed by the supervisors by observing the quality of the interviews, keeping notes and discussing any problems at review sessions. A daily team meeting was mandatory for the survey team. Five percent of the study participants were revisited by the supervisors who administered a short questionnaire consisting of 11 questions focused mainly on identifying any problems in adhering to ethical guidelines and administering questions on particular topics. The second level of monitoring involved cross-checking of completed questionnaires. Each completed questionnaire was cross-checked by another interviewer who shared her observations in the team meeting. At the third level, the QCO rechecked each and every questionnaire on a daily basis, kept notes, provided feedback to the interviewers and supervisors, made corrections where possible, where necessary, guided the interviewer to make a phone call to the respondent to collect any missing data and to correct errors if possible, and sent the interviewer to revisit the respondent if it was necessary. They communicated with the researchers if the problems could not be resolved by themselves and communicated decisions back to the interviewers.

At the fourth level, the research officer and the researchers from icddr,b made frequent field visits, randomly checked filled-out questionnaires, observed interviews where possible and provided feedback to the survey team. Further, a computer-based data checking routine was developed by the icddr,b research team. The data were uploaded to the server every day after the data collection was completed for that day. Due to the efficiency of this system, inconsistencies in the data were identified within a short period of time. The research officer cleaned the data two days before the completion of the data collection in each study area. Problems identified in the data were communicated to the supervisors through the survey coordinator. The supervisor resolved the problems through discussion with the interviewer if possible. If necessary, the interviewer revisited the respondent and solved the issues consulting her. This actually enhanced prompt correction of the data by the QCO based on information received from the field. If the problems could not be resolved using this strategy the researchers were informed and the researchers suggested ways of resolving the issue depending on the nature of the problems.

The similar approach of monitoring was taken to ensure the quality of the management survey data, the only exception was that the survey coordinator was not involved with the monitoring of management data collection.

### Endline survey

Similar strategy from the baseline survey will be followed during the endline survey.

### Cohort tracking

Cohort tracking for end line is essential and therefore participant workers are contacted every 2 months to update essential contact and personal information and details about work, including if she has a new job. Any dropout is also explored to understand the reason. Moreover, attempts will be made to conduct endline interviews with the worker even if she leaves the factory and changes residence within the same area. If she is traced and refuses to be interviewed the reason will be recorded. If she cannot be found in her previous address, we will try to track her through additional contact information. It is assumed that management staff will not move jobs and can be easily contacted through the factory management only the workers are being tracked by icddr,b. Contact information of the study participants is stored separately from the survey data and kept under lock and key.

### Statistical methods: Data analysis

Intention-to-treat (ITT) analysis will be used for assessing the impact of HERrespect. Thus, all the workers selected for the study will be included in the analysis. The primary analysis will involve comparison between intervention and control factories for assessing the impact of full HERrespect intervention (group sessions and factory-wide mobilization) over control. First the data from intervention and control factories and baseline and endline samples will be compared and Chi-square (for categorical variables) and t-tests (for continuous variables) will be performed to test whether there are differences in prevalence of background characteristics. If differences between intervention and control factories and baseline and endline samples are evident, the pre-existing differences will be controlled in subsequent analyses. The impact of HERrespect intervention on main outcomes of interest will be assessed using risk ratios derived from binary regression analyses adjusting for baseline rates. All analyses will be adjusted for the potential factors associated with the outcomes of interest. The same approach will be followed for assessing the secondary outcomes.

### Ethics and dissemination

The study received approval from the Institutional Review Board (IRB) of icddr,b and the South African Medical Research Council Ethics Committee. The participation of the intervention and control factories was based on the factories’ consent to be a part of the study. This study is fully guided by the WHO recommendations for ethical considerations in researching violence against women [35].

Attention was paid when designing the questionnaire to carefully and sensitively introduce and enquire about workers’ experiences of violence and to ensure that questions are framed in a manner that is non-judgmental. There is evidence in the literature that many factory management is opposed to the disclosure of workplace violence and workers disclosing or attempting to address it are victimized through threats, harassment and dismissal [18]. Therefore questions on workplace violence were included in a style which was as far as possible non-threatening to the factory management.

Individual verbal consent was sought by the interviewer prior to the interview with each female garment worker and management staff (see Additional files 3 and 4). All participants were informed orally of the purpose and nature of the study, its expected benefits, sensitivity, confidentiality and voluntary nature of participation. They were asked to give oral consent to participate. Participation in the study was entirely voluntary. The respondents were also free to terminate the interview at any point, and to skip any questions to which s/he did not wish to respond. Written consent was not used due to low levels of readability and concerns regarding confidentiality.

Each interviewed female worker received $6.5 (BDT 500) for participating at the baseline survey. Again at endline $8.5 (BDT 650) will be offered in compensation for their time. The physical safety of interviewees and interviewers from potential retaliatory violence by the perpetrator was of prime importance. If the focus of the research becomes widely known - either within the factory or household or among the wider community it may risk the safety and security of both. Thus, at the factory level the intervention and the study were introduced as focusing on addressing IPV and improving management and in the family and community it was framed as a survey of factory work management and female workers life experiences.

All the study participants were given a unique code and all the indentifying information are kept in a separate file exclusively accessed by the research team and used for tracking the individuals over the intervention period and to contact them during the endline survey. Care will be taken to present the research findings in sufficiently aggregated form to ensure that no participating factories and workers can be identified.

### Limitations

There are constraints on research in the ready-made garment industry in Bangladesh which had to be accommodated in the study design. Factories had strict preference for being included in the study as an intervention or control factory, which the study had to adhere to. This precluded use of a randomisation into the study arms. This means that confounders could not be allocated between arms using randomisation and so have to be measured and adjusted for in analysis where possible. We cannot be sure that all confounders will be measured and adjusted for, but focus on the main possible ones. This will slightly weaken our confidence in the results but having some comparison arm is a strength even if allocation to arm was not random.

We had to take into account the real circumstances of location on the ground and were also not able to match intervention and control factories by location. We have no reason to believe this will affect the results.

In one factory, the number of eligible workers was less than 100. Therefore we recruited all possible eligible workers (77) and relaxed the criteria of duration of employment from 12 to 10 months (23). This is unlikely to impact the results.

The sample (50) for the management survey was selected by participating factories except in one, where the number of management staff was lower than the requirement, so all management were recruited. We recognize that biases may have been introduced in the management sample due to purposive selection conducted by the factories. Any factory approached with such an intervention is likely to use the same strategy of worker and management staff selection. As the intention is to assess impact of an intervention that is feasible to implement and can be scaled up in future, the current sample selection strategy would show the real impact of such intervention.

It is possible that we will find it hard to deliver the full dose of the intervention in factories if managers are reluctant to release workers and managers for periods of 2-3 h for sessions and some simple amendments to sessions may be needed to accommodate available time. This may impact intervention fidelity and ultimately impact.

The primary and secondary outcomes are based on self-reporting and we cannot know if there is under-reporting. This is always a risk in questionnaire-based outcomes and especially where participants may feel the need to protect their family by under-reporting partner violence or may fear reprisals for accurate reporting of workplace violence. We have taken very considerable care around confidentiality, including interviewing women outside work, and hope this will minimise these under-reporting risks.

We have accommodated some loss to follow up in the sample size calculation, but job mobility is a feature of the industry and we are concerned that more women and managers may leave the factories than anticipated. Also any staff leaving intervention factories during the intervention would not get the full dose. Intention to treat requires cohort follow up but mobility may dilute apparent impact. Further absolute loss to follow up will reduce the study’s power to detect the anticipated difference between arms.

## Results

A total of eight factories (four intervention and four control) were recruited for the HERrespect study. A total of 800 female garment workers and 395 management staff were successfully interviewed during the baseline survey.

### Background characteristics of the study sample

Table 3 shows the participants’ background characteristics by study arm. Workers in the intervention factories were generally younger than those in the control factories. In terms of age, 71% of workers from the intervention factories and 58% from the control factories were between 20 and 29 years. In terms of education, workers in the intervention factories were more educated than the control factory workers. In the intervention factories 48% had 6-10 years of education compared to 34% in the control factories. About 24% of the workers from the control factories had no education compared to 14% of those from the intervention factories. In the intervention factories, 54% of workers had been working there for less than 2 years, compared to 20% of those in the control factories. There was no difference in average monthly earnings between arms.

**Table 3:** Comparison between intervention and control groups by background characteristics

Characteristics	Worker survey			Management survey		
	Intervention % (n)	Control % (n)	*P*-value	Intervention % (n)	Control % (n)	*P*-value
N	400	400		195	200	
Age in years						
15-19	8.3	1.3		1.0	0.0	
20-24	36.5	24.8		13.3	2.5	
25-29	34.3	32.8	0.000^a^	25.6	23.5	0.000^a^
30-34	16.3	24.0		30.3	29.5	
≥ 35	4.8	17.8		29.7	44.5	
Level of education						
No education	14.3	23.6		0.0	0.0	
1-5 years	32.0	40.3		3.6	7.0	
6-10 years	48.3	34.3	0.000^a^	48.7	53.8	0.118
11-12 years	4.8	1.8		30.8	21.1	
13-15 years	0.8	0.0		9.7	12.6	
> 15 years	0.8	0.0		7.2	5.5	
Duration of work in the current factory						
0-2 years	53.5	20.0		49.2	13.0	
3-5 years	31.8	43.5	0.000^a^	28.2	22.5	0.000^a^
6 or more	14.8	36.5		22.6	64.5	
Mean earnings per month	8454.6	8555.9	0.290	21,146.5	24,751.1	0.017^b^

^a^Significant at 1% level of significance

^b^Significant at 5% level of significance

As for the workers, the managers in the intervention factories were younger than in the control factories. About 45% of the management staff in the control factories were 35 years or older, compared to 30% of management staff in the intervention factories. Education did not differ significantly between arms. About half of the management staff from both the groups (intervention 49%; control 54%) had 6-10 years of education. Most of the management staff in the intervention factories (49%) had been working there for less than 2 years compared to 13% of those in the control factories. Management staff from the control factories had a greater average monthly income (BDT 24,751 or USD 321) compared to the intervention factories (BDT 21,147 or USD 275).

## Discussion

To our knowledge this is the first study that seeks to evaluate the impact of group sessions with workers, and management; factory-wide campaigns and a community intervention on preventing IPV and workplace violence among female garment workers in Bangladesh.

Literature shows that interventions combining gender transformation group sessions with economic empowerment of women can reduce IPV [21, 23]. However, the majority of these interventions have been done through group-based interventions in Africa, and used approaches for economic empowerment such as microfinance, or jobs and livelihoods training. In contrast to these interventions, HERrespect targets a particularly important group of women, who are already in employment and therefore at some level economically empowered, but potentially exposed to IPV and workplace violence. Thus, the intervention is less complex as it is only focused on gender transformation (as economic empowerment is assumed) and therefore may be effective in a relatively shorter period of time. This intervention could have great potential for scale up across the sector and so is potentially very important.

This study is also novel as it focuses not only on IPV, but also on workplace violence. Studies have shown that female workers face both forms of violence [18], but these are rarely discussed together in literature, nor programmed for simultaneously, despite being driven by similar dynamics of gender inequalities and attempts to control women [18]. This approach allows us to capture a wider picture of experience of violence or poly-victimization by this group of women.

HERrespect programme is a collaborative initiative that brings together the RMG sector buyers (ie., renowned global brands), their suppliers and the local NGOs. Accessing garment factories in Bangladesh for research and/or interventions focusing on VAWG is currently difficult. In this scenario, the strategy of approaching the factories through the buyers proved very effective. A multi-disciplinary team consisting of those experienced in working with the buyers (BSR) and with the factories (Change Associates Ltd.) and in research (icddr,b) strengthened the design and implementation of this study. This model of collaboration is innovative and unique in the field of IPV research, and we suggest is critical for rigorous research in this field.

There are some limitations we faced working in the sector, but we have designed what we perceive as the best possible study within funding and industry constraints. It will provide very important information on the intervention’s effectiveness in this very pragmatic research trial. This research will make valuable contributions to the knowledge and evidence on impact of interventions in a real life situation. Apart from informing programmers and policy makers about intervention effectiveness in reducing IPV and workplace violence against female garment workers this study will also present evidence on an intervention tailored to the garment sector, which makes HERrespect scalable.

## Additional files

Additional file 1: HERrespect worker survey questionnaire. Questionnaire used in the HERrespect baseline survey to collect data from the female garment workers. (PDF 106 kb)

Additional file 2: HERrespect management survey questionnaire. Questionnaire used in the HERrespect baseline survey to collect data from the garment management staff (PDF 52 kb)

Additional file 3: HERrespect worker survey consent form. Consent form used in the HERrespect baseline survey to seek verbal consent from the female garment workers prior to each interview. (PDF 24 kb)

Additional file 4: HERrespect management survey consent form. Consent form used in the HERrespect baseline survey to seek verbal consent from the management staff prior to each interview. (PDF 24 kb)

## Abbreviations

CES-D: Center for Epidemiologic Studies Depression
GEM: Gender equitable men
IPV: Intimate Partner Violence
IRB: Institutional Review Board
ITT: Intention-to-treat
MBI-HSS: Maslach burnout inventory-human service survey
NGO: Non-Government Organisation
PDA: Personalized Digital Assistant
QCO: Quality Control Officer
RMG: Ready-made garment
SAMRC: South African Medical Research Council
VAW: Violence against women
VAWG: Violence against women and girls
WHO: World Health Organization
WPV: Workplace violence
