# Supplementary material for: The HERrespect intervention to address violence against female garment workers in Bangladesh: study protocol for a quasi-experimental trial
Source: BMC Public Health. 2018 Apr 18;18:512. doi: 10.1186/s12889-018-5442-5 (PMC5907290; doi:10.1186/s12889-018-5442-5)
Supplement: Supplementary file 1 — HERrespect worker survey questionnaire. Questionnaire used in the HERrespect baseline survey to collect data from the female garment workers. (PDF 106 kb) [file 12889_2018_5442_MOESM1_ESM.pdf]

DRAFT
Version - 2
HERrespect Evaluation
(ENGLISH)
WORKER SURVEY QUESTIONNAIRE
BASELINE
Facesheet

1. Factory ID	|__|__|__|
2. Worker ID	|__|__|
3. Interviewer ID	|__|__|
4. Name of interviewer	

Household visit form				
	First visit	Second visit	Third visit	
Date<br>Result code *	______________<br>☐☐	______________<br>☐☐	______________<br>☐☐	
Date of next visit	______________	______________	Total visit	☐
Interview start time	☐☐ hr<br>☐☐ min	☐☐ hr<br>☐☐ min	☐☐ hr<br>☐☐ min	
Interview end time	☐☐ hr<br>☐☐ min	☐☐ hr<br>☐☐ min	☐☐ hr<br>☐☐ min	
Total duration of interview	☐☐ hr<br>☐☐ min	☐☐ hr<br>☐☐ min	☐☐ hr<br>☐☐ min	

* Result code<br><br>Complete ....01<br>Refused...02<br>Postponed (mention the reason)<br>______________________..03<br><br>Incomplete ……………………….…………….…04	Household destroyed/ Nobody lives at the house<br>..05<br>Respondent absent ....06<br>Refused to continue the interview (mention the reason) ____________...................................... 07<br><br>Others (mention the reason)<br>_______________...08
SUPERVISOR ID ☐☐	EDITOR ID ☐☐

SECTION 1: BACKGROUND & WORK

The first questions are about yourself, your home and your work situation. Please try and relax, there are no right or wrong answers. Remember that everything you answer will be kept secret.

No.	QUESTIONS AND FILTERS	CODING CATEGORIES	SKIP
101	How old are you?	AGE (YEARS) [ ] [ ]	
102	In which DISTRICT were you born?	NAME OF THE DISTRICT	
103	How long have you lived in this community?	YEARS [ ] [ ]<br>(If less than 1year enter 00)	
104	Have you ever moved or travelled to look for work?	YES……………………………...............................1<br>NO……………………….……...............................2	
105	Can you read and write?	NO.. 0<br>CAN READ ONLY ....1<br>CAN WRITE ONLY ....2<br>CAN READ AND WRITE.................................. ...3	
106	Have you ever attended school/ madrasha or any other institution for study?	YES, SCHOOL..1<br>YES, MADRASHA ....2<br>YES, BOTH...3<br>NO ....4<br>OTHER (Specify) 5	 <br><br>→108<br>→108
107	What is the highest educational level you completed?	CLASS .... [ ][ ]	
108	Are you a member of the following organizations?	Membership: YES / NO	
	a) NGO based microcredit and savings organization	YES 1 / NO 2	
	b) NGO based savings group	YES 1 / NO 2	
	c) Non-NGO savings group	YES 1 / NO 2	
	x) Other NGOs (Specify)	YES 1 / NO 2	
109	What is your current marital status?	CURRENTLY MARRIED, LIVING TOGETHER..1<br>CURRENTLY MARRIED, NOT LIVING TOGETHER..2<br>SEPARATED..3<br>DESERTED..4<br>DIVORCED...5<br>WIDOW..6	(2–6) → END INT.
110	How long have you been married? (Or how long were you married for?)	[ ] YEARS<br>(ENTER 00, IF LESS THAN 1 YEAR)	
111	How many times have you been married?	TIMES [ ]<br>IF MARRIED ONCE GO TO Q 113	
112	Did you have a previous marriage that ended in divorce or widowhood?	WIDOWHOOD…………………………………......1<br>DIVORCE… ……………………….……………......2	
113	Are you related to your husband?	NO……………………………………………………0<br>YES, COUSINS…………………………………......1<br>YES, OTHER RELATIVE…..………………..…….2	
114	How old were you when you married (or were first married, if there has been more than one marriage)?	[ ] AGE IN YEARS	
115	How many children do you have now?	[ ][ ]	

WORK AND MONEY		
The next questions are abotu money and food. Somtimes some family face shortage of money and food. We woulf like to know about your famioly experience.		
116	In the past 4 weeks, how often was there no food to eat of any kind in your house because of a lack of money?	OFTEN..1<br>SOMETIMES...2<br>RARELY..3<br>NEVER..4
117	In the past 4 weeks how often did you or any member of your household go to sleep hungry because of lack of food?	OFTEN..1<br>SOMETIMES...2<br>RARELY..3<br>NEVER..4
118	In the past 4 weeks how often did you or any of your household go a whole day and night without eating because of lack of food?	OFTEN..1<br>SOMETIMES...2<br>RARELY..3<br>NEVER..4
119	How often in the past 4 weeks have you had to borrow food or money because you did not have enough?	EVERYDAY...1<br>MORE THAN ONCE A WEEK ....2<br>ALMOST EVERY WEEK ....3<br>ONCE OR TWICE IN THE LAST 4 WEEKS<br>...4<br>NEVER 5
120	If you had an emergency at home and needed 50,000 Tk, how easy would you say it would be to find the money?	VERY DIFFICULT.. 1<br>SOMEWHAT DIFFICULT....................................2<br>FAIRLY EASY...3<br>VERY EASY 4
121	Considering all the money you earned from work or any other sources (such as selling things), how much did you earn last month?	_______________ TK
122	How many Tk did yousave in the last 4 weeks?	Tk _______________<br>(If none write 00)
123	How many Tk have you got in savings?	Tk_______________<br>(If none write 00)

SECTION 2: WORK AND STRESS			
201	For how long you have been working in the current factory?	[ ] [ ] year [ ] [ ] months	
202	What position do you hold in the factory?	HELPER.......................1<br>JUNIOR SEWING OPERATOR.............................2<br>OPERATOR...3<br>SENIOR SEWING OPERATOR ....4<br>QUALITY INSPECTOR...5<br>REPORTER...6<br>PRODUCTION REPORTER..................................7<br>FUSING HELPER..8<br>FUSING OPERATOR...9<br>FOLDING MAN..10<br>FOLDING OPERATOR..11<br>CUTTER MAN..12<br>NIDDLE MAN...13<br>PACKING MAN..14<br>HOUSE KEEPING..15<br>CANTEEN GIRL...16<br>IRON GIRL...17<br>ISSUE GIRL..18<br>SAMPLE ASST..19<br>SPOT MAN...20<br>OTHER (SPECIFY..21	
203	How long you have been working in this position?	[ ] [ ] year [ ] [ ] months	
204	Did you worked in other garment factories previously?	YES ....1<br>NO 2	→ 206
205	In total, how many garment factories have you worked in (including the current one)?	[ ] [ ]	
206	For how long you have been working in the garment sector?	[ ] [ ] year [ ] [ ] months	
207	Did you ever have any occupation other than garment work?	YES ....1<br>NO 2	
208	On average how much do you earn per month?	TK _________________	
209	In that last three months, have you ever not been paid your proper monthly wage?	YES ....1<br>NO 2	
210	In the last three months, have you been allowed time off work for medical appointments or domestic problems?	YES, I WANTED AND IT WAS GRANTED..........1<br>WANTED AND MOST OF TIME IT WAS GRANTED..2<br>WANTED AND MOST OF TIME IT WAS REFUSED..3<br>WNATED AND NEVER GRANTED......................4<br>HALF OF THE TIME IT WAS GRANTED AND HALF OF THE TIME IT WAS REFUSED............5<br>NO I WANTED TO ASK BUT DID NOT ASK ....6<br>I DID NOT NEED IT 7	
211	Do you have a letter of appointment?	YES ....1<br>NO 2	
212	What is the share of your contribution to your household income?	FULL (100) ....1<br>MORE THAN HALF..2<br>HALF...3<br>LESS THAN HALF...4	

213	Would you say that the money that you bring into the family is more than what your husband contributes, less than what he contributes, or about the same as he contributes?	MORE THAN HUSBAND..1<br>LESS THAN HUSBAND..2<br>ABOUT THE SAME ....3<br>HUSBAND PAYS ALL ....4<br>I PAY ALL 5			
214	How long have you been earning an income?	[ ] [ ] YEARS			
215	The following statements are a series of statements about your current work situation. Please say if you strongly agree, agree, disagree or strongly disagree with these phrases:	STRONGLY DISAGREE	DISAGREE	AGREE	STRONGLY AGREE
	a) You are frequently stressed or depressed because of not having enough income	1	2	3	4
	b) You are frequently stressed or depressed because you are not proud of what you do to get money	1	2	3	4
	c) You are frequently stressed or depressed becauseyou want or have to help your family with money	1	2	3	4

SECTION 3: HEALTH AND WELLBEING

We would now like to ask you some questions about your health and well-being.

The next few questions are about how you have been feeling in the past week. Each question is a statement; please answer how many days you have had particular feelings or ideas or whether you have not had them at all. There are four options: rarely or never, some or a little of the time, a moderate amount of time, or most or all of the time.

301	CES-D SCALE	RARELY OR NONE OF THE TIME	SOME OR A LITTLE OF THE TIME (1-2 DAYS)	MODERATE AMOUNT OF TIME (3-4 DAYS)	MOST OR ALL OF THE TIME (5-7 DAYS)
A	During the past week you were bothered by things that usually don't bother you	0	1	2	3
B	During the past week you did not feel like eating, your appetite was poor	0	1	2	3
C	During the past week you felt you could not cheer yourself up even with the help of family and friends	0	1	2	3
D	During the past week you felt you were just as good as other people	0	1	2	3
E	During the past week you had trouble keeping your mind on what you were doing	0	1	2	3
F	During the past week you felt depressed	0	1	2	3
G	During the past week you felt that everything you did was an effort	0	1	2	3
H	During the past week you felt hopeful about the future	0	1	2	3
I	During the past week you thought your life had been a failure	0	1	2	3
J	During the past week you felt fearful	0	1	2	3
K	During the past week your sleep was restless	0	1	2	3
L	During the past week you were happy	0	1	2	3
M	During the past week you talked less than usual	0	1	2	3
N	During the past week you felt lonely	0	1	2	3
O	During the past week people were unfriendly	0	1	2	3
P	During the past week you enjoyed life	0	1	2	3
Q	During the past week you had crying spells	0	1	2	3
R	During the past week you felt sick	0	1	2	3
S	During the past week you felt that people dislike you	0	1	2	3
T	During the past week you could not get 'going'	0	1	2	3
302	In the past four weeks, has the thought of ending your life been in your mind?	YES)……………………………………………………1<br>NO………………………………………………………2			

Next we would like to ask you about how you feel about yourself generally.

I'll read out a list of statements about your general feelings about yourself. Please say whether you strongly agree, agree, disagree or strongly disagree with the following statements

303	**Rosenberg Self-Esteem Scale**	STRONGLY DISAGREE	DISAGREE	AGREE	STRONGLY AGREE
A	On the whole, you are satisfied with yourself	1	2	3	4
B	At times, You think you are no good at all	1	2	3	4
C	You feel that you have a number of good qualities	1	2	3	4
D	You are able to do things as well as most other people	1	2	3	4
E	You feel you do not have much to be proud of	1	2	3	4
F	You certainly feel useless at times	1	2	3	4
G	You feel that you are a person of worth, at least on an equal plane with others	1	2	3	4
H	You wish to have more respect for yourself	1	2	3	4
I	All in all, you are inclined to feel that you are a failure	1	2	3	4

J	You take a positive attitude toward yourself	1	2	3	4	
304	The following statements are series of statements about how you see your life now:	STRONGLY DISAGREE	DISAGREE	NEITHER AGREE OR DISAGREE	AGREE	STRONGLY AGREE
	a) In most ways your life is close to your ideal	1	2	3	4	5
	b) The conditions of your life are excellent	1	2	3	4	5
	c) You are satisfied with your life	1	2	3	4	5
	d) So far you have got the important things you wanted in life	1	2	3	4	5

SECTION 4 ATTITUDES ABOUT RELATIONS BETWEEN MEN AND WOMEN

The next sets of questions are about your views on life and particularly on relations between men and women in society. There are no right or wrong answers – we are just interested in what you think.

Now, I'll read out some statements. Please say whether you strongly agree, agree, disagree or strongly disagree with each of the statements

401		STRONGLY DISAGREE	DISAGREE	AGREE	STRONGLY AGREE
A	A woman's most important role is to take care of her home and cook for her family	1	2	3	4
B	Men need sex more than women do	1	2	3	4
C	There are times when a woman deserves to be beaten	1	2	3	4
D	It is a woman's responsibility to avoid getting pregnant	1	2	3	4
E	A woman should tolerate violence in order to keep her family together	1	2	3	4
F	I think that a woman should obey her husband	1	2	3	4
G	I think that a woman needs her husband's permission to do paid work	1	2	3	4
H	I think that a woman cannot refuse to have sex with her husband	1	2	3	4
I	I think if a woman does not physically fight back, it's not rape	1	2	3	4
J	I think that men should share the work around the house with women such as doing dishes, cleaning and cooking	1	2	3	4
K	I think that children belong to a man and his family	1	2	3	4
L	I think that if a wife does something wrong her husband has the right to punish her	1	2	3	4
402	In your opinion, does a man have good reason to hit his wife if –				
	a) She does not complete her household work to his satisfaction	1	2	3	4
	b) She disobeys him	1	2	3	4
	c) She refuses to have sexual relations with him	1	2	3	4
	d) She asks him whether he has other girlfriend	1	2	3	4
	e) He suspects that she is unfaithful	1	2	3	4
	f) He finds out that she has been unfaithful	1	2	3	4

SECTION 5 CURRENT HUSBAND			
**No.**	**QUESTIONS & FILTERS**	**CODING CATEGORIES**	**SKIP TO**
501	I would now like you to tell me a little about your <u>current</u> husband.<br><br>How old is your husband?<br>PROBE: MORE OR LESS	AGE (YEARS).................... [ ][ ]	
502	Do you and he both have the same level of education or do you have more schooling or does he have more schooling?	SAME …………………......................1<br>YOU ARE MORE EDUCATED ....2<br>HE IS MORE EDUCATE …………......3	
503a	Does your husband drink alcohol? (EVER)	YES..1<br>NO..2<br>DO NOT KNOW....................................3	(2, 3) → 506
503	During the past 12 months, how often does your husband drink alcohol?<br><br>1. Most days<br>2. Weekly<br>3. Once a month<br>4. Less than once a month<br>5. Never<br>(Probe : if she says she doesn't know – ask how often she has known that he has had a drink of alcohol)	MOST DAYS ....1<br>WEEKLY..2<br>ONCE A MONTH..3<br>LESS THAN ONCE A MONTH.....................4<br>NEVER..5<br>REFUSED/NO ANSWER............................9	(5) ⇒**506**
504	In the <u>past 12 months</u>, how often have you seen your husband drunk? Would you say most days, weekly, once a month, less than once a month, or never?	MOST DAYS...1<br>WEEKLY..2<br>ONCE A MONTH..3<br>LESS THAN ONCE A MONTH.....................4<br>NEVER..5<br>REFUSED/NO ANSWER................................9	
505	In the <u>past 12 months</u>, have you experienced any of the following problems, related to your husband drinking?<br>a) Money problems<br>b) Family problems<br>x) Any other problems, specify	YES / NO<br>a) MONEY PROBLEMS 1 2<br>b) FAMILY PROBLEMS 1 2<br>x) OTHER 1 2	
506	Does your husband ever use drugs? Would you say:<br><br>1. Nearly every day<br>2. 1–3 times a week<br>3. 1–3 times a month<br>4. Occasionally, less than once a month<br>5. In the past, not now<br><br>(Probe : if she says she doesn't know – ask how often she has known that he has used drugs)	NEARLY EVERY DAY.................................1<br>1–3 TIMES A WEEK...2<br>1 – 3 TIMES IN A MONTH............................3<br>LESS THAN ONCE A MONTH.....................4<br>NEVER...5<br>IN THE PAST, NOT NOW.............................6<br>REFUSED/NO ANSWER...............................9	
507	In the <u>past 12 months</u>, he ever been involved in a physical fight with another man? Has this happened once or twice, a few times, many times or never?	NEVER..1<br>ONCE OR TWICE...2<br>A FEW (3-5) TIMES..3<br>MANY (MORE THAN 5) TIMES...................4<br>DON'T KNOW /DON'T REMEMBER...........8	
508	Has your <u>current</u> husband had a relationship with any other women while being with you?	YES ....1<br>NO...2<br>MAY HAVE..3	

509	Does your husband have any other wives while being married (having a relationship) with you?	YES ....1<br>NO...2<br>DON'T KNOW/DON'T REMEMBER............8	
510	How many wives does he have (including yourself)?	NUMBER OF WIVES [ ][ ]<br><br>DON'T KNOW/DON'T REMEMBER..........98	
511	Has your <u>current</u> husband had children with any other woman while being with you?	YES..1<br>NO...2<br>MAY HAVE..3<br>DON'T KNOW /DON'T REMEMBER...........8	

SECTION 6: INTIMATE RELATIONSHIP

When two people marry, they usually share both good and bad moments. I would now like to ask you some questions about your current relationships and how your husband treats you. If anyone interrupts us I will change the topic of conversation. I would again like to assure you that your answers will be kept secret, and that you do not have to answer any questions that you do not want to. May I continue?

No.	QUESTIONS & FILTERS	CODING CATEGORIES	SKIP TO
601	In your relationship with your current husband, how often would you say that you quarrelled? Would you say rarely, sometimes or often?	NEVER....1<br>RARELY ....2<br>SOMETIMES....3<br>OFTEN....4	

The next set of statements are about your relationship with your **current husband**, please say for each if you strongly agree, agree, disagree or strongly disagree:

602	RELATIONSHIP CONTROL SCALE	STRONGLY DISAGREE	DISAGREE	AGREE	STRONGLY AGREE
	a) When he wants sex he expects you to agree	1	2	3	4
	b) If you asked him to use a condom, he would get angry	1	2	3	4
	c) He won't let you wear certain things	1	2	3	4
	d) He has more to say than you do about important decisions that affect both of you	1	2	3	4
	e) He tells you who you can spend time with	1	2	3	4
	f) When you wear things to make you look beautiful he thinks you may be trying to attract other men	1	2	3	4
	g) He wants to know where you are all of the time	1	2	3	4
	h) He lets you know you are not the only partner he could have	1	2	3	4
	i) He gets angry with you when you are late home from the factory	1	2	3	4
	j) He threatens to stop allowing you to work	1	2	3	4

The next few questions are things that your may have experienced from your **current husband**.

603	In the past 12 months, how many times your current husband has	NEVER	ONCE	FEW TIMES	MANY TIMES
	a) Prohibited you from getting a job, going to work, trading, earning money or participating in income generation activities?	0	1	2	3
	b) Took your earnings, jewellery or any valuable things from you against your will?	0	1	2	3
	c) Refused to give you money you needed for household expenses even when he has money for other things (such as alcohol and cigarettes)?	0	1	2	3
	d) Threw you out of house?	0	1	2	3
	e) Did not work despite his capacity to earn?	0	1	2	3
	f) Insisted you to surrender your earnings partially or fully either to him or to an in-law?	0	1	2	3
	g) Did not allow you to spend your own earnings without his permission.	0	1	2	3

The next questions are about things that happen to many women, and that your **current husband** may have done to you.

		NEVER	ONCE	FEW TIMES	MANY TIMES
604	In the past 12 months, how many times your current husband has				
	a) Insulted you or made you feel bad about yourself?	0	1	2	3
	b) Belittled or humiliated you in front of other people?	0	1	2	3
	c) Done things to scare or intimidate you on purpose (e.g. by the way he looked at you, by yelling and smashing things)?	0	1	2	3
	d) Threatened to hurt you?	0	1	2	3
	e) Hurt people you care about as a way of hurting you, or damaged things of importance to you?	0	1	2	3

Some women find these questions hard to answer, others easy. Remember everything you share here will only be used for research purposes and will be kept secret. We are interested now in your relationship with **current husband.**

		NEVER	ONCE	FEW TIMES	MANY TIMES
605	In the past 12 months, how many times your current husband has				
	a) Slapped you or thrown something at you that could hurt you?	0	1	2	3
	b) Pushed you or shoved you or pulled your hair?	0	1	2	3
	c) Hit you with his fist or with something else that could hurt you?	0	1	2	3
	d) Kicked, dragged, beaten, choked or burnt you?	0	1	2	3
	e) Threatened with or actually used a gun, knife or other weapon against you?	0	1	2	3

CHECK 6A	Check if - 1 or 2 or 3 is marked in Q605 (a-e) .... 1<br>Check - if 0 is marked in all Q605 (a-e) 2	
	The next few questions are about things you may have experienced with your **current husband**	
606	How would you describe your sexual relationship with your current husband?	VERY SATISFYING……………………..….1<br>SATISFYING……………………………...2<br>UNSATISFYING………….........................3<br>VERY UNSATISFYING...........................4

		NEVER	ONCE	FEW TIMES	MANY TIMES
607	a) In the past 12 months, how many times your current husband has physically forced you to have sexual intercourse when you did not want to?	0	1	2	3
	b) In the past 12 months, how many times has your current husband used threats or intimidation to get you to have sex when you did not want to?	0	1	2	3
	c) In the past 12 months, how many times did you have sexual intercourse you did not want to because you were afraid of what your husband might do?	0	1	2	3
	d) In the last 12 months, how many times current husband has forced you to do something sexual that you found degrading or humiliating?	0	1	2	3
	e) In the last 12 months, how many times your current husband has forced you to watch pornography when you did not want to?	0	1	2	3

CHECK 6B	Check if - 1 or 2 or 3 is marked in Q604 (a-e) .... 1<br>Check - if 0 is marked in all Q604 (a-e) 2	
CHECK 6C	WOMAN EXPERIENCED PHYSICAL OR SEXUAL VIOLENCE (1 circled in CHECK 6A OR CHECK 6B)..1<br><br>WOMAN HAS NOT EXPERIENCED ANY PHYSICAL OR SEXUAL VIOLENCE ONLY (2 circled in both CHECK 6A and CHECK 6B...2 →	609

608	Did you go to any of the following for help during last 12 months	YES	NO	
	a) Relatives from partner's side	1	2	
	b) Relatives from own side	1	2	
	c) Neighbours	1	2	
	d) Someone at work	1	2	
	e) Friends	1	2	
	f) Police/court	1	2	
	g) Hospital or health centre	1	2	
	h) Local leader	1	2	
	i) NGOs (specify)	1	2	
	x) Anywhere else? (Specify)	1	2	
609	Do you know from where one can get help for such experience? If yes, where?<br><br>MARK ALL THAT APPLY.	DON'T KNOW.................................A<br>POLICE..B<br>LAWYER.......................................C<br>COURT...D<br>DOCTOR..E<br>ONE STOP SERVICE CENTRE ...<br>..F<br>VICTIM SUPPORT CENTER(..G<br>HELP LINE NO..............................H<br>NGO (Specify) ....I<br>LOCAL LEADER...........................J<br>OTHER (Specify) X		

SECTION 7 OTHER EXPERIENCES

In their lives, many women have unwanted experiences and experience different forms of maltreatment and violence from all kinds of people, men or women. These may be relatives, other people that they know, and/or strangers. If you don't mind, I would like to ask you about some of these situations. Everything that you say will be kept confidential.

No.	QUESTIONS & FILTERS	CODING CATEGORIES	SKIP TO
701	**Since the age of 15 until now, has anyone other than your** husband ever **forced you into sexual intercourse** when you did not want to, for example by threatening you, holding you down, or putting you in a situation where you could not say no. Remember to include people you have known as well as strangers. Please at this point exclude **attempts** to force you.<br><br>IF NECESSARY: We define sexual intercourse as vaginal, oral or anal penetration.<br><br>NOTE THAT THIS QUESTIONS IS ABOUT RAPES THAT ACTUALLY HAPPENED	YES ....1<br>NO..2	 → **704**
**702**	During the past 12 months, how many times **has anyone other than your** husband ever **forced you into sexual intercourse** when you did not want to?	NEVER...1<br>ONCE..2<br>FEW TIMES.......................................3<br>MANY TIMES....................................4	
**703**	Who did this to you?<br>PROBE: Anyone else? How about a relative? How about someone at school or work? How about a friend or neighbour? A stranger or anyone else?<br>***DO NOT READ OUT THE LIST. MARK LETTER FOR ALL MENTIONED.***		
	FATHER	A	
	FATHER-IN-LAW	B	
	BROTHER	C	
	OTHER FAMILY MEMBER	D	
	COUSIN	E	
	OTHER RELATIVE	F	
	FAMILY MALE FREIND	G	
	NEIGHBOUR (MALE)	H	
	SOMEONE AT WORK (MALE)	I	
	FRIEND/ACQUAINTANCE (MALE)	J	
	RECENT ACQUAINTANCE (MALE)	K	
	COMPLETE STRANGER (MALE)	L	
	TEACHER (MALE)	M	
	DOCTOR/HEALTH STAFF (MALE)	N	
	RELIGIOUS LEADER	O	
	POLICE/ SOLDIER (MALE)	P	
	OTHER (specify) _____________	X	

No.		A.		B.IF YES: Has this happened in the past 12 months?		
**704**	**Since the age of 15 until now**, any of the following has happened to you? Remember to include people you have known as well as strangers.	YES	NO	YES	NO	
	a) Has anyone **attempted but NOT succeed to force** you into sexual intercourse when you did not want to, for example by holding you down or putting you in a situation where you could not say no?	1	2	1	2	
	b) Touched you sexually. This includes for example touching of breasts or private parts?	1	2	1	2	
	c) Made you touch their private parts against your will	1	2	1	2	

CHECK7A	**AT LEAST ONE 'YES' ('1') MARKED IN COLUMN A. [ ]** ⇓ 705	**ONLY 'NO' ('2') MARKED** **[ ]⇒**	⇒ **706**

705	Who did this to you?<br><br>PROBE:<br>Anyone else? How about a relative? How about someone at school or work? How about a friend or neighbour? A stranger or anyone else?<br>***DO NOT READ OUT THE LIST***<br>***MARK LETTER FOR ALL MENTIONED***		
	FATHER	A	
	FATHER-IN-LAW	B	
	BROTHER	C	
	OTHER FAMILY MEMBER	D	
	COUSIN	E	
	OTHER MALE RELATIVE	F	
	FAMILY MALE FREIND	G	
	NEIGHBOUR (MALE)	H	
	SOMEONE AT WORK (MALE)	I	
	FRIEND/ACQUAINTANCE (MALE)	J	
	RECENT ACQUAINTANCE (MALE)	K	
	COMPLETE STRANGER (MALE)	L	
	TEACHER (MALE)	M	
	DOCTOR/HEALTH STAFF (MALE)	N	
	RELIGIOUS LEADER	O	
	POLICE/ SOLDIER (MALE)	P	
	OTHER (specify) ____________	X	
706	Have you ever been asked to perform sexual acts or tolerate sexual touching against your will in order to get a job or keep your job, or to get promoted?	YES ....1<br>NO ....2<br>REFUSED/NO ANSWER................9	
707	Have you been asked to perform sexual acts or tolerate sexual touching against your will at work in the last 12 months?	YES ....1<br>NO ....2<br>REFUSED/NO ANSWER................9	

There are many cases where your supervisor asks you to do something and you do it, even though you may not see clearly why it should be done. We are interested in finding out why you might do as your supervisor asks, so I will give you some possible reasons and would like you to tell me how likely each of these reasons is.

708	**Social power scale**	Very unlikely	Somewhat likely	Very likely
	**REWARD**			
A	If you do not comply with your supervisor, you will not be rewarded	1	2	3
B	The only reason for doing as supervisor suggests is to obtain good things in return	1	2	3
C	You want to do as supervisor suggests only because of the good things s/he will give you for complying	1	2	3
D	Your supervisor has the ability to reward you (in some manner) if you do as s/he suggests	1	2	3
E	If you do not do as supervisor suggests you will not receive good things from her/him	1	2	3
F	In this situation you are dependent on supervisor 's willingness to grant you good things	1	2	3
	**COERCION**			
G	Supervisor can harm you in some manner if you do not do as s/he suggests	1	2	3
H	If you do not do as supervisor suggests, s/he will punish you	1	2	3
I	Something bad will happen to you if you don't do as supervisor requests and s/he finds out.	1	2	3
J	You had better do as supervisor suggests in order to prevent something bad from happening to me.	1	2	3

K	Supervisor might do something which is unpleasant to those we do not do as s/he suggests	1	2	3
	**LEGITIMATE**			
L	It is your duty to comply with supervisor	1	2	3
M	Because of supervisor 's position he has the right to influence your behaviour	1	2	3
N	You are obligated to do as supervisor suggests	1	2	3

**709**	How often within the past 4 weeks have you experienced or witnessed a manager:	Never	Once	2-3 Times	Many Times
A	Call a worker names	0	1	2	3
B	Report a worker to some one more senior for punishment	0	1	2	3
C	Scold a worker	0	1	2	3
D	Make fun of a workerbecause of their appearance	0	1	2	3
E	Make fun of a workerfor some other reason	0	1	2	3
F	Shout a worker	0	1	2	3
G	Slap a worker	0	1	2	3
H	Strike the head of a worker	0	1	2	3
I	Pull the hair of a worker	0	1	2	3
J	Push or shove a worker	0	1	2	3
K	Praise a workeror a line that was working very well	0	1	2	3
L	Discuss with a workerproblems at home which make it hard for her to do overtime and found a solution for both of you	0	1	2	3
M	Lowered production targets for a day after realising that there was particular difficulty in achieving them	0	1	2	3
N	Discuss with the workerhow best to organize the work so that targets can be met	0	1	2	3
O	Review the production targets to make sure they can be achieved in normal working hours	0	1	2	3

710	The next questions ask about difficulties you may have doing certain activities because of a HEALTH PROBLEM or injury you have had	No difficulty	Yes Some difficulty	Yes A lot of difficulty	Cannot do at all
A	Do you have difficulty seeing, even if wearing glasses?	1	2	3	4
B	Do you have difficulty hearing?	1	2	3	4
C	Do you have difficulty walking or climbing steps ?	1	2	3	4
D	Do you have difficulty remembering or concentrating?	1	2	3	4
E	Do you have any difficulty washing all over or dressing?	1	2	3	4
F	Do you have difficulty speaking?	1	2	3	4

711	Please tell me if you own any of the following, either by yourself or with someone else:	NO	YES Own by self	YES Own with others	YES Own by self and with others
	a) Land	1	2	3	4
	b) House	1	2	3	4
	c) Business	1	2	3	4
	d) Large household items (TV, bed, cooker)	1	2	3	4
	e) Jewellery, gold or other valuables	1	2	3	4
	f) Savings in the bank	1	2	3	4
	x) Other property, specify	1	2	3	4

SECTION 8 COMPLETION OF INTERVIEW			
801	We have now finished the interview. Do you have any comments, or is there anything else you like to add?		
802	I have asked you about many difficult things.<br>How has talking about these things made you felt?	GOOD/BETTER ....1<br>BAD/WORSE...................................2<br>SAME/ NO DIFFERENCE.................3	
	FINISH ONE – IF RESPONDENT HAS DISCLOSED PROBLEMS / VIOLENCE<br><br>I would like to thank you very much for helping us. I appreciate the time that you have taken. I realise that these questions may have been difficult for you to answer, but it is only by hearing from women themselves that we can really understand about their health and experiences of violence.<br><br>From what you have told us, I can tell that you have had some very difficult times in your life. No-one has the right to treat someone else in that way. However, from what you have told me I can see that you are strong, and have survived through some difficult circumstances.<br><br>***FINISH TWO - IF RESPONDENT HAS NOT DISCLOSED PROBLEMS / VIOLENCE***<br><br>I would like to thank you very much for helping us. I appreciate the time that you have taken. I realise that these questions may have been difficult for you to answer, but it is only by hearing from women themselves that we can really understand about women's health and experiences in life.		
**INTERVIEWER COMMENTS TO BE COMPLETED AFTER INTERVIEW**			
