# Supplementary material for: The HERrespect intervention to address violence against female garment workers in Bangladesh: study protocol for a quasi-experimental trial
Source: BMC Public Health. 2018 Apr 18;18:512. doi: 10.1186/s12889-018-5442-5 (PMC5907290; doi:10.1186/s12889-018-5442-5)
Supplement: Supplementary file 2 — HERrespect management survey questionnaire. Questionnaire used in the HERrespect baseline survey to collect data from the garment management staff (PDF 52 kb) [file 12889_2018_5442_MOESM2_ESM.pdf]

HERrespect Evaluation

BASELINE SURVEY

MANAGEMENT SURVEY QUESTIONNAIRE
(ENGLISH)

Facesheet

1. Factory ID	|__|__|
2. Manager ID	|__|__|__|
3. Interviewer ID	|__|__|
4. Name of interviewer	

Visit form			
	First visit	Second visit	Third visit
Date<br><br>Result code *	____________<br>☐☐	____________<br>☐☐	____________<br>☐☐
Date of next visit			Total visit ☐
Interview start time	☐☐ hr<br>☐☐ min	☐☐ hr<br>☐☐ min	☐☐ hr<br>☐☐ min
Interview end time	☐☐ hr<br>☐☐ min	☐☐ hr<br>☐☐	☐☐ hr<br>☐☐
Total duration of interview	☐☐ hr<br>☐☐ min	☐☐ hr<br>☐☐ min	☐☐ hr<br>☐☐ min

* Result code

Complete 01
Refused 02
Postponed (mention the reason)
______________________............................03

Incomplete ………………….. ……………….04

Respondent absent 05
Refused to continue the interview
______________... 06

Others (mention the reason)
______________...07

SUPERVISOR ID ☐☐	EDITOR ID ☐☐

SECTION 1: BACKGROUND INFORMATION						
QUESTIONS & FILTERS		CODING CATEGORIES				SKIP TO
101	How old were you on your last birthday? (MORE OR LESS)	AGE (YEARS) ....|__|__|				
102	What is the highest class you completed at school?	CLASS |__|__|				
103	Are you currently studying?	YES .... 1<br>NO 2				
104	Have you done a training course that has given you a certificate or diploma?	YES .... 1<br>NO 2				
105	Are you currently married or have you been married?	CURRENTLY MARRIED………………………...1<br>DIVORCED …..........……………….……….……2<br>WIDOWED ………....…………………….…......3<br>NEVER MARRIED ……………………………….4				
106	How much do you earn per month? (Approximately)	TK |__|__||__|__||__|__|				
107	If a person became ill in your home and Tk. 100,000 was needed for treatment or medicine, how easy would you say it would be to find the money?	VERY DIFFICULT..1<br>SOMEWHAT DIFFICULT……………………......2<br>SOMEWHAT EASY..3<br>VERY EASY……………………………….....…4				
108	Of your total household expenses, what proportion is met through your own earnings?	None..1<br>Very little...2<br>Some...3<br>Half.................... ....4<br>Most...5<br>All...6				
109	The following statements are series of statements about how you see your life now:	STRONGLY DISAGREE	DISAGREE	NEITHER AGREE OR DISAGREE	AGREE	STRONGLY AGREE
	a) In most ways your life is close to your ideal	1	2	3	4	5
	b) The conditions of your life are excellent	1	2	3	4	5
	c) You are satisfied with your life	1	2	3	4	5
	d) So far you have got the important things you wanted in life	1	2	3	4	5

SECTION 2: WORK AND BURNOUT			
201	For how long you have been working in the current factory?	[ ] [ ] year [ ] [ ] months	
202	What position do you hold?	Junior supervisor ....1<br>Supervisor...2<br>Senior supervisor ....3<br>Line chief ....4<br>Quality controller..........................5<br>Quality in-charge ....6<br>Quality auditor ....7<br>Cutting assistant ....8<br>Cutting in charge ....9<br>Assistant production manager (A.P.M)..10<br>Production manager ....11<br>Finishing in charge.........................12<br>Packing in charge ....13<br>I.E officer...14<br>Floor in-charge...............................15<br>Assistant general manager (A.G.M)................................16<br>General manager (G.M) ....17<br>Other_____________...........18<br>(specify)	
203	How long you have been working in this position in the current factory?	[ ] [ ] year [ ] [ ] months	
204	How many female workers do you supervise directly?	[ ] [ ] Female workers	
205	How many male workers do you supervise directly?	[ ] [ ] Male workers	
206	Have you worked in other garment factories?	YES ....1<br>NO 2	→ 208
207	In total, how many garment factories have you worked in (including the current one)?	[ ] [ ]	
208	For how long you have been working in the garment sector?	[ ] [ ] years	

209	The following statements are a series of statements about your current work situation. Please say if you strongly agree, agree, disagree or strongly disagree with these phrases:	STRONGLY DISAGREE	DISAGREE	AGREE	STRONGLY AGREE
	a) You are frequently stressed or depressed because of not having enough work	1	2	3	4
	b) You are frequently stressed or depressed because of not having enough income	1	2	3	4
	c) You are frequently stressed or depressed because you are not proud of what you do to get money	1	2	3	4
	d) You are frequently stressed or depressed because you want or have to help my family with money	1	2	3	4

The next questions we would like to ask are about work, accomplishment. Below is a list of statements dealing with your feeling about work. Each question is a statement; please answer how frequently you have had particular feelings or ideas or whether you have not had them at all.

210	**Maslach Burnout Inventory (MBI)**	Never	A few times year	Monthly	A few times a month	Every Week	A few times a week	Every day
	**Emotional Exhaustion**							
A	You feel emotionally drained from your work	0	1	2	3	4	5	6
B	You feel used up at the end of the workday	0	1	2	3	4	5	6

C	You feel fatigued when you get up in the morning and have to face another day on job	0	1	2	3	4	5	6
D	Working with people all day is really a strain for you	0	1	2	3	4	5	6
E	You feel burned out from your work	0	1	2	3	4	5	6
F	You feel frustrated by your job	0	1	2	3	4	5	6
G	You feel you are working too hard on your job	0	1	2	3	4	5	6
H	Working with people directly puts too much stress on you	0	1	2	3	4	5	6
I	You feel like you are at the end of your rope	0	1	2	3	4	5	6
	**Personal accomplishment**							
J	You can easily understand how workers who you supervise feel about things	0	1	2	3	4	5	6
K	You deal very effectively with the problems of your supervisees	0	1	2	3	4	5	6
L	You feel you are positively influencing other people's life through your work	0	1	2	3	4	5	6
M	You feel very energetic	0	1	2	3	4	5	6
N	You can easily create a relaxed atmosphere with your supervisees	0	1	2	3	4	5	6
O	You feel exhilarated after working closely with your supervisees	0	1	2	3	4	5	6
P	You have accomplished many worthwhile things in this job	0	1	2	3	4	5	6
Q	In your work, you deal with emotional problems very calmly	0	1	2	3	4	5	6
	**Depersonalization**							
R	You feel you treat some supervisees as if they were impersonal 'objects'	0	1	2	3	4	5	6
S	You have become more callous toward people since you took this job	0	1	2	3	4	5	6
T	You worry that this job is hardening you emotionally	0	1	2	3	4	5	6
U	You don't really care what happens to some of your supervisees	0	1	2	3	4	5	6
V	You feel your supervisees blame you for some of their problems	0	1	2	3	4	5	6
	**Optional items**							
W	You feel similar to your supervisees in many ways	0	1	2	3	4	5	6
X	You feel personally involved with your supervisees problem	0	1	2	3	4	5	6
Y	You feel uncomfortable about the way you have treated some of your supervisees	0	1	2	3	4	5	6

SECTION 3 ATTITUDES ABOUT RELATIONS BETWEEN MEN AND WOMEN

The next set of questions are about your views on life and particularly on relations between men and women in society. There are no right or wrong answers – we are just interested in what you think.

For each of the following statements please say answer whether you strongly agree, agree, disagree or strongly disagree with the following statements:

301		STRONGLY DISAGREE	DISAGREE	AGREE	STRONGLY AGREE
A	A woman's most important role is to take care of her home and cook for her family.	1	2	3	4
B	Men need sex more than women do.	1	2	3	4
C	There are times when a woman deserves to be beaten.	1	2	3	4
D	It is a woman's responsibility to avoid getting pregnant.	1	2	3	4
E	A woman should tolerate violence in order to keep her family together.	1	2	3	4
F	If someone insults a man, he should defend his reputation, with force if he has to.	1	2	3	4
G	To be a man, a person needs to be tough.	1	2	3	4
H	A man should have the final say in decisions in his home.	1	2	3	4
I	I think that a woman should obey her husband	1	2	3	4
J	I think that a woman needs her husband's permission to do paid work	1	2	3	4
K	I think that a woman cannot refuse to have sex with her husband.	1	2	3	4
L	I think if a woman does not physically fight back, it's not rape	1	2	3	4
M	I think that there is nothing a woman can do if her husband wants to have girlfriends	1	2	3	4
N	I think that men should share the work around the house with women such as doing dishes, cleaning and cooking	1	2	3	4
O	I think that children belong to a man and his family	1	2	3	4
P	I think that if a man beats you it shows that he loves you	1	2	3	4

302	Who in your household usually has the final say regarding the health of women in the family? Would you say yourself, your partner or both equally?	YOURSELF ....1<br>WIFE/PARTNER ....2<br>BOTH EQUALLY...3<br>OTHER MEMBER OF HOUSEHOLD<br>...4<br>N/A 5	
303	Who in your household usually has the final say about decisions involving your children (their schooling, their activities)? Would you say yourself, your partner or both equally?	YOURSELF ....1<br>WIFE/PARTNER...2<br>BOTH EQUALLY.......................................3<br>OTHER MEMBER OF HOUSEHOLD<br>..4<br>N/A...5	
304	Who has the final say about decisions involving how your family spends money on food and clothing? Would you say yourself, your partner or both equally?	YOURSELF...1<br>WIFE/PARTNER ....2<br>BOTH EQUALLY..3<br>OTHER MEMBER OF HOUSEHOLD<br>..4<br>N/A..5	
305	Who has the final say about decisions involving how your family spends money on	YOURSELF..1<br>WIFE/PARTNER 2	

	large investments such as buying a car, a house or a household appliance?	BOTH EQUALLY ....3<br>OTHER MEMBER OF HOUSEHOLD<br>..4<br>N/A...5	

	**MANAGEMENT ATTITUDES**				
306	For each of the following statements please say whether you strongly agree, agree, disagree or strongly disagree with the following statements	STRONGLY DISAGREE	DISAGREE	AGREE	STRONGLY AGREE
A	Sewing operators usually want to do as little work as possible	1	2	3	4
B	Male supervisors are much better at meeting production targets than women would be	1	2	3	4
C	Sewing operators should have their pay reduced if they don't work hard enough	1	2	3	4
D	Women supervisors are too soft on women workers	1	2	3	4
E	You need to be tough to be a supervisor or manager	1	2	3	4
F	If they haven't finished work Sewing operators should just stay until the targets are completed	1	2	3	4
G	Sewing operators in a garment factory should always obey their supervisor and managers	1	2	3	4
H	It's not your concern if women Sewing operators get into trouble at home for staying late to meet targets	1	2	3	4

307	STRATEGIES TO ENSURE PRODUCTION TARGETS ARE MET<br>Supervisors, line chiefs and managers are often placed under a lot of pressure to meet production targets. Below is a list of things that some supervisors and managers do to try and get the workers to work more quickly. How often have you done these in the last four weeks? would you say never, once, 2-3 times or many times.				
	How often within the past 4 weeks have you	Never	Once	2-3 Times	Many Times
A	Called a sewing operator or helper names	0	1	2	3
B	Reported a sewing operator or helper to some one more senior for punishment	0	1	2	3
C	Scolded a sewing operator or helper	0	1	2	3
D	Made fun of a sewing operator or helper because of their appearance	0	1	2	3
E	Made fun of a sewing operator or helper for some other reason	0	1	2	3
F	Shouted a sewing operator or helper	0	1	2	3
G	Slapped a sewing operator or helper	0	1	2	3
H	Struck the head of a sewing operator or helper	0	1	2	3
I	Pulled the hair of a sewing operator or helper	0	1	2	3
J	Pushed or shoved a sewing operator or helper	0	1	2	3
K	Praised a sewing operator or a line that was working very well	0	1	2	3
L	Discussed problems at home with a sewing operator which make it hard for her to do overtime and found a solution for both of you	0	1	2	3
M	Lowered production targets for a day after you realized that there was particular difficulty in achieving them	0	1	2	3
N	Discussed with the sewing operators and helpers how best to organize the work so that targets can be met	0	1	2	3
O	Review the production targets with your management and make sure they were feasible in normal working hours	0	1	2	3

SECTION 4: KNOWLWDGE AND ATTIDUES REGARDING LAWS AND POLICIES						
**No.**	**QUESTIONS & FILTERS**	**CODING CATEGORIES**				**SKIP TO**
401	Do women have higher or equal or lower rights compared to men according to Bangladesh constitution?	HGHER....1<br>EQUAL....2<br>LOWER….3				
402	Are there laws/policies in this country that protect women from discrimination?	YES ….1<br>NO ……….2<br>DON'T KNOW……….3				
403	Are there laws/policies in this country that protect women against spousal violence?	YES ….1<br>NO ……2<br>DON'T KNOW 3				 → 405<br>→ 405
404	With regards to these laws about violence against women, do you strongly agree, agree, disagree or strongly disagree with the following statements	STRONGLY AGREE	AGREE	NO OPINION	DISAGREE	STRONGLY DISAGREE
a	They make it too easy for a woman to bring a violence charge against a man	1	2	3	4	5
b	These laws are too harsh	1	2	3	4	5
c	These laws are not harsh enough	1	2	3	4	5
d	They do not provide enough protection for the victim of violence	1	2	3	4	5
405	Are there laws/policies in this country that protect women against workplace violence?	YES ….1<br>NO ..….2<br>DON'T KNOW 3				 → 407<br>→ 407
406	The policy address which of the following violence<br>a) Physical violence b) Economic violence c) Emotional violence<br>d) Sexual violence e) None<br><br>MARK ALL THAT APPLY.	PHYSICAL VIOLENCE ....A<br>ECONOMIC VIOLENCE ....B<br>EMOTIONAL VIOLENCE ....C<br>SEXUAL VIOLENCE ....D<br>NONE ....E<br>DON'T KNOW....F				
407	Do you strongly agree, agree, disagree or strongly disagree with the statements: "Sexual remarks and gesture is an act of workplace violence".	1	2	3	4	5

SECTION 5 COMPLETION OF INTERVIEW			
501	We have now finished the interview. Do you have any comments, or is there anything else you like to add?		
502	I have asked you about many difficult things. How has talking about these things made you feel?	GOOD/BETTER....1<br>BAD/WORSE....2<br>SAME/ NO DIFFERENCE 3	
	FINISH<br>I would like to thank you very much for helping us. I appreciate the time that you have taken. I realise that these questions may have been difficult for you to answer, but it is only by hearing from person like you we can really understand about work experiences and related stress.		
**INTERVIEWER COMMENTS TO BE COMPLETED AFTER INTERVIEW**			
