# Supplementary material for: The HERrespect intervention to address violence against female garment workers in Bangladesh: study protocol for a quasi-experimental trial
Source: BMC Public Health. 2018 Apr 18;18:512. doi: 10.1186/s12889-018-5442-5 (PMC5907290; doi:10.1186/s12889-018-5442-5)
Supplement: Supplementary file 3 — HERrespect worker survey consent form. Consent form used in the HERrespect baseline survey to seek verbal consent from the female garment workers prior to each interview. (PDF 24 kb) [file 12889_2018_5442_MOESM3_ESM.pdf]

Consent Form for female garment workers

Protocol No.16036	Version No. 1.00	Date:

Protocol Title: **Evaluation of HERrespect: an intervention for addressing factory work management and female workers life experiences**

Investigator's name: Dr. Ruchira Tabassum Naved_________________________________

Organization: __icddr,b____

Purpose of the research
Assalamuwalaikum/Adab, my name is ..... I work for "Cholera hospital" (icddr,b). You are invited to take part in a research study by taking part in an interview. "Cholera hospital" (icddr,b) is conducting this study to learn more about the family and work experiences of female garment workers involved with "HERrespect programme".

Why invited to participate in the study?
You have been chosen to participate in the study, as a group member of the "HERrespect programme" in which your factory is participating. . By talking to you we expect to learn a lot about family and work experiences of female workers of readymade garment (RMG) factories in Bangladesh. This interview is a part of "HERrespect programme" evaluation.

Methods and procedures [What is expected from the participants of the research study?]
If you agree to participate in this study, we would like to interview you in a private setting of your choice, where you will feel comfortable to talk with us. During the interview, you will be asked questions about your experience of within family and workplace. Overall, we would like to know about your socio-economic status, marriage, health, marital relationship, family experience and work experience.

How long will the interview last?
The interview will take about an hour and a half. If necessary we might also request you for a repeat interview. The follow-up interview may be conducted to verify the accuracy of our understanding of the information you offer or to elicit further information or to cross-check information gathered from other sources. You will hold the right to decide whether to participate in that interview. We also wish to re-interview you after 18 months with similar types of questions and expect to last for an hour and a half.

Risk and benefits
You may find it difficult to answer one or more questions that we ask. However, most of the participants in similar studies appreciated the opportunity to talk about such experiences. There is no direct benefit, in material terms, to you for participating in the study. If you participate, we will be benefited as it will help us and the policy makers to learn about the female garment workers family and life experiences. This understanding is important for coming up with appropriate interventions and policies.

Privacy, anonymity and confidentiality
The information that is collected from you will be kept private and confidential. The study team will make every effort to protect your privacy and maintain the confidentiality of all the information that you provide. Your name or other identifiers will not be appeared in any results or reports of the study. Data will be password protected that only the study team can access.

Future use of information
Your name and address will only be used in follow up studies for reaching you and only upon your informed consent you will be included in those studies. In case of use of the information collected

from this study by other researchers, only anonymous or abstracted information and data may be supplied.

Right not to participate and withdraw
Your participation in this study is completely voluntary. If you decide not to participate, you will not lose any existing benefits to which you are entitled. If you agree to participate in this study, you may end your participation at any time without penalty or loss of existing benefits to which you are entitled. Even if you decide to take part, you are free to refrain from answering any questions that makes you uncomfortable. You are also free to withdraw at any time without affecting benefits and services you might avail from icddr,b.

Principle of compensation
You will receive **BDT 500 for this interview and BDT 650 at the time of re-interview after 18 months** as a compensation of **your time.**

Do you have any questions? [Please note down the question, and respond]
Do you agree to be interviewed?

NOTE WHETHER THE INFORMANT AGREES TO PARTICIPATE IN THE STUDY OR NOT AND AGREES TO HAVING THE SESSION TAPE RECORDED

[ ] DOES NOT AGREE TO PARTICIPATE → THANK FOR HER TIME & END

[ ] AGREES TO PARTICIPATE → THANK FOR AGREEING TO PARTICIPATE

Please tell me if this time_____________ and place________________ are good to talk?

If there are any problems we can agree on a place and time of your choice.

What if I need more information?
If you have any questions about this study, you may ask them to me now. If you think of any questions later, please contact Dr. Ruchira Tabassum Naved (880-2-9827001-10; Ext: 2234, ruchira@icddrb.org).

What if there is a problem?
If you have any questions about your rights in this study, please contact Mr. M.A. Salam Khan (9886498 or 8860523-32, ext. 3206), the icddr,b Committee Coordination Secretariat.

Investigator or person who conducted Informed Consent discussion: I, the undersigned, have explained to the volunteer in a language she understands the procedures to be followed in the study and the potential risks and benefits involved, and, confidentiality of personal information. I confirm that she has agreed to participate in the study after being fully informed. I promise to keep information provided by the participant private and confidential.

Name of person obtaining consent:____

Signature of person obtaining consent: _______________________________Date:____________

Signature of witness: ____Date:____________
